# A Study of the Effects of Virtual Reality-Based Sports Games on Improving Executive and Cognitive Functions in Minors with ADHD—A Meta-Analysis of Randomized Controlled Trials

**DOI:** 10.3390/bs14121141

**Published:** 2024-11-28

**Authors:** Weixiao Zhang, Haojie Li, Yi Sheng

**Affiliations:** 1 School of Athletic Performance, Shanghai University of Sport, Shanghai 200438, China; weixiao.zhang@student.uts.edu.au (W.Z.);; 2 Business School, University of Technology Sydney, Sydney, NSW 2007, Australia; 3 School of Exercise and Health, Shanghai University of Sport, Shanghai 200438, China

**Keywords:** ADHD, cognitive function, executive function, children’s mental health, virtual motor games

## Abstract

(1) Background: Attention Deficit Hyperactivity Disorder (ADHD) is a common mental health condition in children that can significantly impact their quality of life. In this study, we compared the effectiveness of virtual reality (VR) technology with traditional rehabilitation training through meta-analysis, aiming to provide a basis for the clinical optimization of rehabilitation strategies. (2) Methods: The study was registered in PROSPERO, and a search was conducted using the subject terms “virtual reality” and “attention deficit hyperactivity disorder” across six databases. The search yielded 10 randomized controlled trials (RCTs) that met the inclusion criteria. The data were analyzed using a random-effects model in statistical software. (3) Results: The study found that VR exercise game technology significantly outperformed the control group in terms of both the primary outcome (executive function and cognitive function) and secondary outcomes (attention, memory, and task switching) in children under the age of 18 with ADHD (children under 18 years of age are defined as adolescents and children). Sensitivity analyses confirmed the robustness of the five outcome measures. Bias tests revealed no publication bias for the primary outcome, but some bias for the secondary outcomes, which did not affect the overall results. (4) Conclusion: VR motor games significantly improved the executive and cognitive functions of children with ADHD.

## 1. Introduction

Attention Deficit Hyperactivity Disorder (ADHD) is a prevalent mental health problem whose prevalence has been steadily increasing, and ADHD occurs not only in children, but also in adolescents and adults, posing an increasing public health challenge worldwide [1,2]. Research suggests that this rise in prevalence may not solely be attributed to an actual increase in cases, but also to improvements in diagnostic methods and heightened public awareness. As awareness of ADHD continues to expand, healthcare professionals have become more adept at identifying the disorder, leading to a greater recognition of affected individuals. Additionally, the increased awareness of ADHD symptoms among parents and educators has facilitated earlier diagnosis and intervention for many children [3,4]. The substantial changes observed in these statistics point to a genuine rise in ADHD prevalence. ADHD is defined in the DSM-5 as a neurodevelopmental disorder primarily characterized by inattention, hyperactivity, and impulsivity. The ICD-11 definition of ADHD is largely consistent with that of the DSM-5, emphasizing inattention and hyperactivity as core features. These symptoms are persistent and have a profound impact on an individual’s daily functioning. Both definitions highlight the multifaceted nature of ADHD and its significant effect on an individual’s quality of life [5]. Individuals with ADHD exhibit deficits in inhibitory control, which can hinder their ability to regulate behavior and lead to impulsive actions. These impulsive behaviors can, in turn, contribute to the development of more severe mental health issues [6].

Task switching refers to the ability to transition quickly between different tasks, while inhibiting impulses involves the capacity to suppress or control automatic responses or impulses to avoid inappropriate or disadvantageous behaviors. These cognitive functions enable individuals to effectively manage and regulate their behavior in response to complex environmental and task demands. Cognitive functions, broadly speaking, encompass a range of abilities, including attention and memory, which form the foundation for an individual’s capacity to acquire knowledge, solve problems, and engage in social interactions [7]. Current research on minors under the age of 18 with ADHD primarily focuses on the incidence and symptomatology, academic and social impacts, as well as the research methodologies and assessment tools employed [8,9]. Lee HK et al. (2014) demonstrated that minors with ADHD exhibit significant deficits in both executive and cognitive functioning, which in turn affect their learning abilities, social skills, and daily life [10]. Irwin et al. (2021) found that children with ADHD show significantly lower working memory capacity compared to their typically developing peers [11] and also exhibit reduced abilities in multitasking and information updating. These cognitive deficits not only impair learning ability but may also contribute to a decrease in self-efficacy, potentially creating a vicious cycle. Moreover, cognitive deficits in ADHD patients are often linked to their emotional and behavioral difficulties [12]. In studies of executive functioning, Arnold et al. (2020) reported that minors with ADHD struggle with planning, organization, and task switching [13], frequently experiencing difficulties in completing homework, which results in declining academic performance [14] and may ultimately lead to school dropout [15]. The working memory capacity of ADHD patients is notably lower than that of their non-ADHD peers, directly affecting their ability to process and apply information in learning contexts [16,17,18]. Allan et al. (2019) further emphasized that deficits in cognitive and executive functions in minors with ADHD exacerbate over time, leading to increasingly detrimental effects [19]. Therefore, it is crucial to explore effective interventions aimed at improving executive and cognitive functioning in minors with ADHD. Although a substantial body of research has highlighted cognitive and executive deficits in ADHD patients, there are still notable limitations in existing studies, particularly regarding the evaluation of intervention outcomes and long-term effects. Consequently, further investigation into effective interventions remains a central focus of current research.

Previous studies have primarily focused on the treatment of ADHD through three main approaches, listed as follows. (1) Medication intervention: the use of methamphetamine, methylphenidate, and amphetamines, as demonstrated in the study from Jaffe et al. (2005), has been shown to significantly improve attention and executive functioning in individuals with ADHD [20]; (2) psychological and behavioral interventions: Virone (2021) indicates that behavioral interventions—such as parent training, school-based interventions, integrated programs combining medication and behavioral strategies, and cognitive training combined with behavioral therapies—are effective in enhancing working memory and attention in ADHD patients, thereby improving their learning outcomes and overall quality of life [21]; (3) exercise interventions: for instance, interventions involving aerobic exercise, team sports, yoga, martial arts, dance, outdoor adventure activities, swimming, and cycling have proven effective in improving attention and self-control in individuals with ADHD [22,23]. However, these traditional interventions still face limitations in terms of enhancing engagement and enjoyment and reducing side effects.

With the advancement of artificial intelligence (AI) technologies, some studies have begun exploring the integration of technological interventions for ADHD. However, existing interventions still lack personalization and interactivity. Consequently, there is a growing need to investigate newer methods that can improve intervention efficacy and increase children’s active participation. Virtual reality (VR) sports gaming technology represents an emerging intervention modality that blends elements of sports and gaming to offer an immersive experience [24,25]. Current research demonstrates that VR sports games can enhance executive and cognitive functions in children with ADHD through various modalities, including VR training, virtual running, interactive sports games, virtual teamwork exercises, virtual adventures, sports simulations, memory training, and contextual simulations [26,27,28]. Furthermore, sensor-based devices, such as motion capture sensors, accelerometers, and gyroscopes, are widely utilized in these VR-based movement interventions. For example, the virtual dance game using Kinect sensors in the study by Rodrigues et al. (2018) successfully captured children’s movements in real time, providing immediate feedback and boosting their sense of engagement [29]. Similarly, virtual running applications employing accelerometers were able to adjust game difficulty based on children’s motor performance, which helped improve both their motor skills and attention span. Utilizing VR motion gaming technology to enhance the executive and cognitive functions of children with ADHD offers several advantages, including heightened engagement, real-time feedback, increased motivation, and multisensory stimulation, all of which contribute to improved cognitive functions. Additionally, the personalized settings within VR motion gaming technology allow for interventions tailored to each child’s individual needs, promoting more effective and adaptive treatment outcomes.

The innovation of this study is the first systematic research on the effectiveness of using virtual reality racing game technology as an intervention. In addition, the study will systematically assess the specific effects of virtual reality sports games on cognitive and executive functions of ADHD patients by means of a randomized controlled trial to provide a scientific basis for clinical practice. Through this study, we hope to provide new ideas and methods for the intervention of ADHD patients, promote research progress in related fields, and provide practical solutions for the rehabilitation and social adaptation of minor ADHD patients. The paper is structured as follows: Section 2 introduces the research methodology and data processing procedures, Section 3 describes in detail the data analysis and presentation of the results, Section 4 presents a discussion of the findings, and finally, Section 5 summarizes the results of the study and makes recommendations for future research.

## 2. Methods

### 2.1. Protocol and Registration

The PRISMA statement is a set of guidelines intended to improve the quality of systematic reviews and meta-analyses. It covers areas such as literature searches, selection criteria, data extraction, and result presentation. The core objective of PRISMA is to ensure transparency and reproducibility of research, thereby enhancing the credibility of research results. Using the PRISMA statement involves conducting a comprehensive literature search based on the development of inclusion and exclusion criteria to ensure the relevance of the selected studies to the research objective. Studies that meet the criteria are selected, and the rationale for decisions is documented throughout the selection process. This process usually includes initial screening, full-text review, and final inclusion, ensuring methodological consistency throughout. This systematic review is based on the Preferred Reporting Items for Systematic Reviews and Meta-Analyses (PRISMA) guidelines and is registered with the International Prospective Systematic Review Registry (PROSPERO) website. The registration number is CRD42024581533 [30].

### 2.2. Sources of Information

A search was performed in WOS, PubMed, Embase, Cochrane, EBSCO, CNKI up to August 2024, and the results were extracted into Endnote.

### 2.3. Search Strategy

The search strategy limited the language to English and Chinese and to all documents published before August 2024. In searching for relevant literature on the impact of virtual reality motion gaming technology on people with ADHD, as shown in Table 1, the relevant subject terms used in relation to the full search strategy in Cochrane were (a) Virtual Reality, Virtual OR Virtual Reality, Educational OR Educational Virtual Realities OR Virtual Realities, Educational OR VR OR Video Games, and (b) Attention Deficit Disorder with Hyperactivity OR ADHD OR ADDH OR Attention Deficit Disorders with Hyperactivity OR Attention Deficit Hyperactivity Disorders OR Hyperkinetic Syndrome OR Or Minimal Brain Dysfunction.

### 2.4. Inclusion and Exclusion Criteria

The inclusion criteria were summarized based on the PICOS methodology used in this study. The PICOS methodology included population, intervention, comparison, outcome, and study design.

The literature review inclusion criteria were as follows: (1) subjects were all ADHD minors under the age of 18, regardless of gender; (2) the intervention was about virtual reality motor gaming technology, e.g., (computer games, computer programming); (3) the study had to compare the intervention with a control group or with a control group; (4) the endpoint indicator assessed at least one of the phases of cognitive and executive functioning of minors with ADHD; (5) the study had to be a randomized controlled trial for quality reasons; and (6) the research language had to be Chinese or English.

The exclusion criteria for the literature review were as follows: (1) studies that were not randomized controlled trials; (2) studies that did not concern minor ADHD patients; (3) studies that did not provide explicit data on statistical values (e.g., mean and standard deviation); (4) studies for which the full text of the paper could not be found; (5) interventions that were not about virtual reality sports gaming technology; and (6) research studies not in Chinese or English.

### 2.5. Data Extraction and Synthesis

Data extraction involved three processes, listed as follows. 1. Study selection: A systematic literature search strategy was used to search the databases to ensure that relevant fields were covered. Clear inclusion and exclusion criteria were then formulated to determine which studies should be included and which should be excluded. 2. Data extraction: the data extracted included the author and publication year, sample size, source of the study, participant characteristics, intervention characteristics, research indicators and measurement tools, and research results. 3. Literature quality assessment: The quality of each study was assessed using the Cochrane risk of bias tool. The quality assessment results for each study were then recorded in a data extraction table for subsequent analysis.

Data synthesis mainly included four aspects, listed as follows. 1. Effect size calculation: according to the type of analysis results, it was finally determined to be a continuous variable, so the standardized mean difference (SMD) or mean difference (MD) was selected as the effect size index. Then, the effect size calculation and synthesis were performed using Stata statistical software 18. 2. Heterogeneity testing: the I^2^ value was calculated to assess the heterogeneity between different studies. The higher the I^2^ value, the greater the heterogeneity. 3. Model selection: due to the strong heterogeneity between the individual studies, a random-effects model was finally selected. 4. Sensitivity analysis: the impact of these studies on the overall results was assessed by performing sensitivity analyses on five outcome indicators to ensure the robustness of the results. The entire ‘extraction and synthesis’ process is the core of a meta-analysis. Systematic steps and rigorous methods can effectively integrate and evaluate the results of existing studies and then draw more reliable conclusions.

### 2.6. Study Quality Assessment

Research quality assessment is an important step in ensuring the reliability of meta-analysis results. The Cochrane Intervention System Review Handbook was finally selected for research quality assessment. Selection bias, performance bias, detection bias, attrition bias, and reporting bias were recorded. Two evaluations independently rated the bias of each study as high, low, or unclear. If articles were rated as low, they were considered to be of high methodological quality, and if articles were rated as high, they were considered to be of low methodological quality. In the Cochrane Handbook for Systematic Reviews of Interventions, risk of bias is assessed as low, unclear, or high. Low risk means the study used an appropriate method of randomization, all participants received the same intervention and the outcome assessors were blinded, and there was no selective reporting, indicating that the results of the study are credible. Unclear means there is insufficient information in the study about randomization, blinding, or outcome reporting to determine whether there is a risk of bias, so the assessment result is unclear. High risk means the study failed to use randomization, the blinding was not properly implemented or only some of the results were reported, and there is obvious selection bias or other sources of bias [31]. When making an overall judgement on the article, studies with a low risk in all areas are classified as high-quality literature. When there is a high risk of bias in at least one area, the overall judgement is one of low-quality literature. All others are considered to be medium-quality literature. If there was any disagreement, a third-party reviewer was involved in order to reach a consensus. Based on the above criteria, we found that the ten included studies comprised four high-quality studies, three moderate-quality studies, and three low-quality studies. Overall, the included literature met our criteria. By conducting this assessment, we ensured the quality of the included studies and thus improved the credibility of the meta-analysis results.

### 2.7. Statistical Analysis

The meta-analysis was performed in this study using a random-effects model using Stata software 18, the effect size was calculated as follows. 1. Selecting the effect size: for continuous data, we used the mean difference (MD) and the standardized mean difference (SMD). 2. Data extraction: we extracted the required data such as sample size, mean value, and standard deviation from each study to ensure the accuracy of the information. 3. Calculating the effect size: we used the appropriate statistical formula to calculate the effect size of the study and calculate its standard error (SE) accordingly. 4. Combining effect sizes: using the random-effects model, we combined the effect sizes and standard errors of each study to calculate the overall effect size. The random effects model takes into account heterogeneity between studies. Effect sizes were calculated using the mean and standard deviation of the outcome variables. The I^2^ statistic was used to assess the heterogeneity of the included studies, with less than 50% indicating low heterogeneity, 50–75% indicating moderate heterogeneity, and more than 75% indicating high heterogeneity. If heterogeneity (I^2^ > 50%) was present, sensitivity analyses were used to investigate the sources of heterogeneity and to assess the stability of the overall results by excluding individual studies. The sensitivity analysis was performed as follows. 1. Defining the purpose of the sensitivity analysis: a sensitivity analysis aims to assess the impact of specific assumptions or methods on the results; The robustness of the results can be tested by varying certain parameters. 2. Implementation methods involved the following steps. (1) Excluding specific studies; we excluded certain studies one by one and observed the change in the overall effect size to assess the impact of individual studies on the results. (2) We also changed the effect size calculation method. Different effect size calculation methods can be tried to check whether the results are consistent. (3). We then assessed heterogeneity through comparing results by adjusting the t-statistic model to see the effect of heterogeneity on the final conclusion. 3. Reporting the results. We clearly presented the results of the sensitivity analysis, including how the analysis was performed and its effect on the overall effect, so that readers can understand the robustness of the analysis. To verify the publication bias of the studies, funnel plots representing the relationship between sample size and effect size were used.

## 3. Results

### 3.1. Study Selection

**Figure 1:**
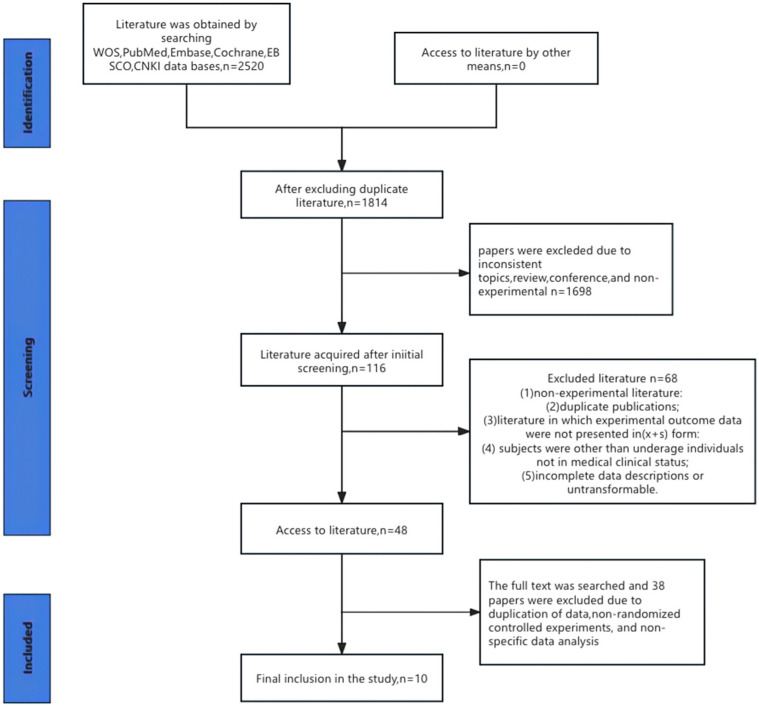
Flow chart of literature screening.

A total of 2520 articles related to the topic of this study were searched from six databases, including WOS, PubMed, Embase, Cochrane, EBSCO, and CNKI; 1814 articles remained after duplicates were excluded by using Endnote21 software. After screening layer by layer, a total of 10 RCTs were finally included in this study. The selection process and the final included studies are visually represented in Figure 1, which illustrates the flow of article selection, including the number of articles screened, excluded, and ultimately included in this analysis.

### 3.2. Basic Characteristics of the Included Literature

This study ultimately included ten papers, as summarized in Table 2. The collective research involved a total of 683 subjects from six different countries, providing a diverse participant base. Among these subjects, 332 were assigned to the intervention group, while the remainder constituted the control group. The study population had a mixed gender profile, with participants ranging in age from 6 to 17 years old. The interventions employed in these studies were categorized into three primary types: 1. computer-based training, 2. motor games, and 3. virtual reality (VR) training. Notably, the specific intervention programs varied significantly across the studies, highlighting the diversity in approaches to treatment. The duration of each intervention session ranged from 15 to 65 min, with the majority of sessions clustering around 30 min, indicating a common preference for shorter, focused training periods. In terms of frequency, the number of intervention sessions varied widely, from as few as zero sessions to as many as six sessions, suggesting a range of implementation strategies among the studies. Finally, the intervention period also varied greatly, with the shortest period being 4 weeks and the longest period being 20 weeks.

### 3.3. Methodological Assessment of the Included Literature

**Figure 2:**
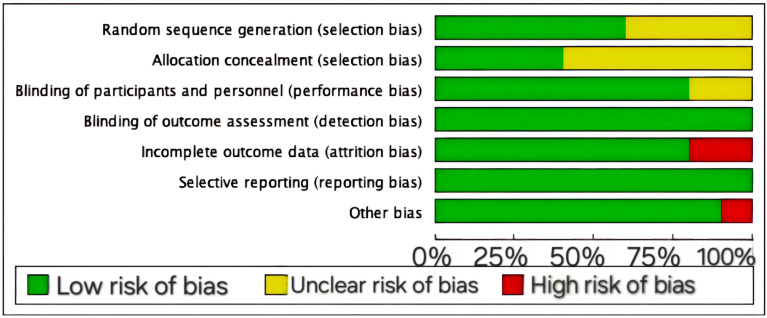
Risk-of-bias graph.

**Figure 3:**
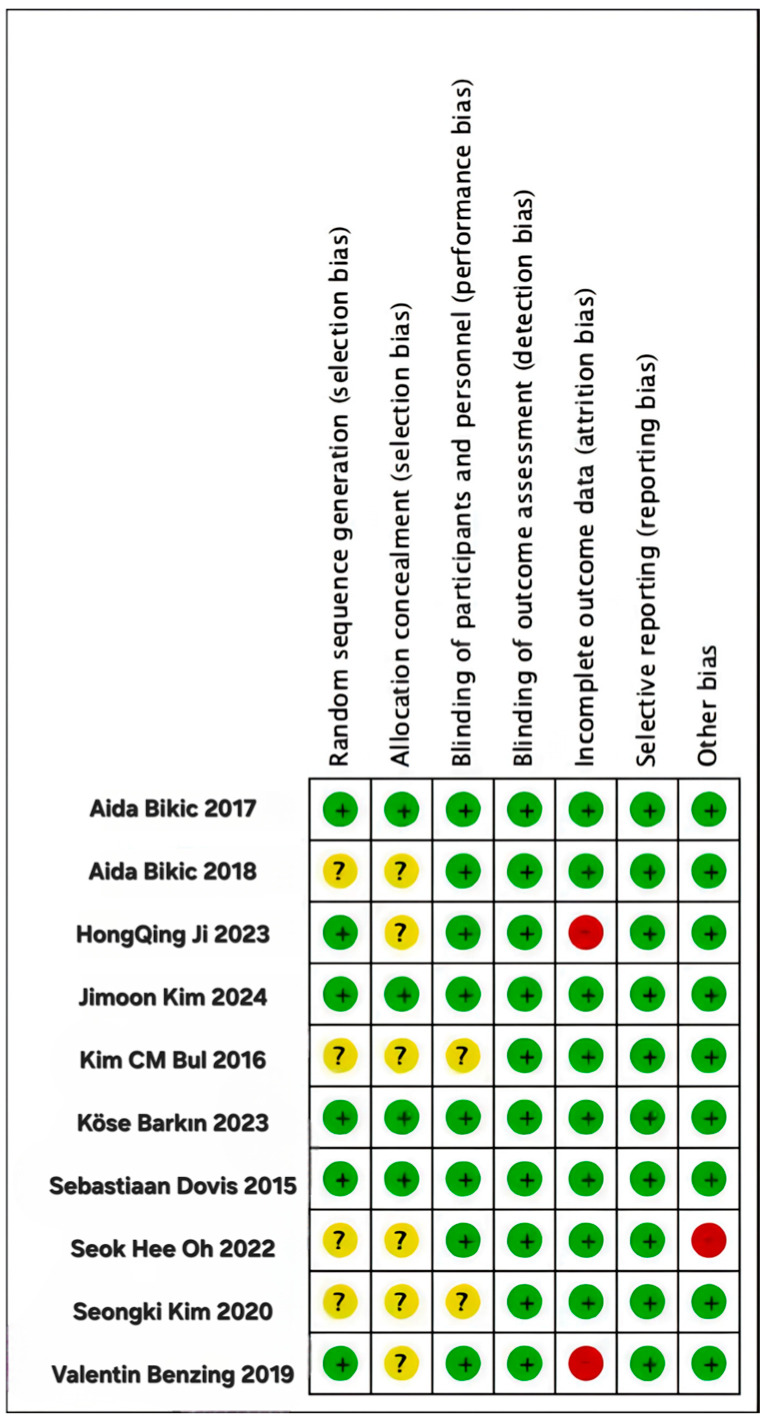
Risk of bias summary.

Table 3 presents the quality scores for the 10 studies according to the Cochrane Handbook for Systematic Reviews of Interventions. These scores provide a systematic assessment of methodological rigor and overall quality. According to the established criteria for assessing the quality of research, 4 of the 10 studies were classified as high-quality literature, indicating that they followed sound research methods and were highly reliable. Three studies were considered to be of moderate quality. Three studies were also classified as low-quality literature. The results of the assessment are presented visually in Figure 2 and Figure 3, which illustrate the distribution of quality scores for the included studies. These graphs help to understand the overall quality of the studies and to quickly and visually compare the studies.

### 3.4. Analysis of Outcomes

#### 3.4.1. Main Outcome Indicators

##### Effects of Virtual Reality Exercise Technology on Cognitive Function in Minor ADHD Patients

**Figure 4:**
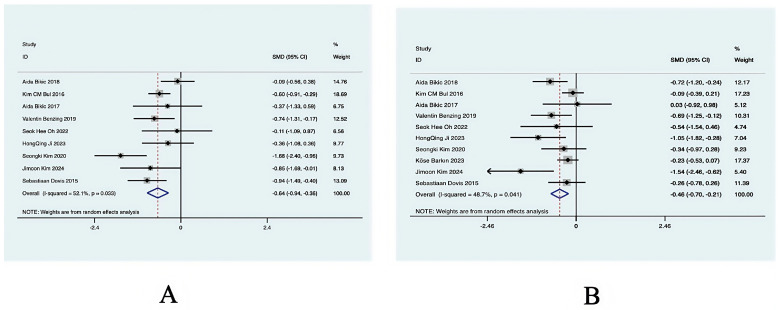
Forest diagram of key indicators: (**A**) cognitive function and (**B**) executive function.

Figure 4A shows the effect of virtual reality exercise technology on cognitive function in minor ADHD patients. The article included nine RCT studies, which found (SMD = −0.64, 95% CI (−0.94, −0.35), *p* = 0.033, I^2^ = 52.1%) moderate heterogeneity in the overall effect, which was statistically significant; a random-effects model was used to analyze the effect size. The results of the study suggest that virtual reality exercise technology has an improving effect on cognitive function in minor ADHD patients.

##### Effects of Virtual Reality Movement Technology on Executive Functioning in Minor ADHD Patients

Figure 4B shows the effects of virtual reality movement technology on executive function in minor ADHD patients. The article included 10 RCT studies, which found (SMD = −0.46, 95% CI (−0.7, −0.21), *p* = 0.031, I^2^ = 48.7%) low heterogeneity in the overall effect, which was statistically significant; a random-effects model was used to analyze the effect size. The results of the study suggest that virtual reality exercise technology has an improving effect on executive function in minor ADHD patients.

#### 3.4.2. Secondary Outcome Indicators

##### Effect of Virtual Reality Exercise Technology on Attention in Minor ADHD Patients

**Figure 5:**
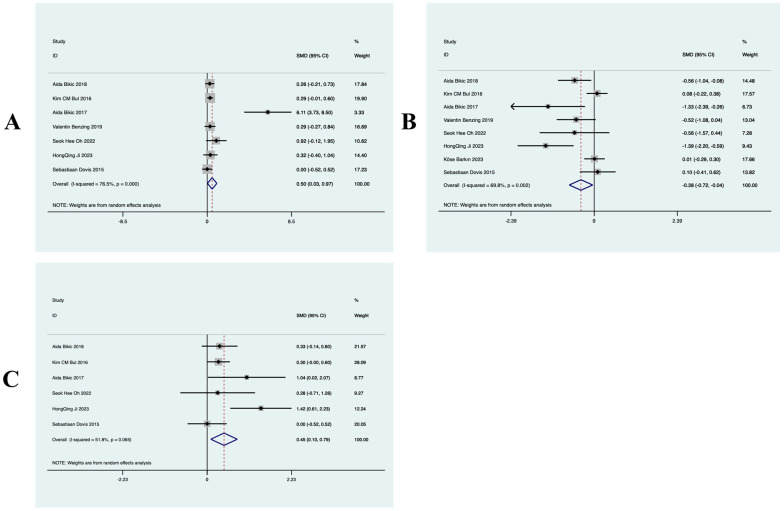
Forest plot of secondary indicators (**A**) is attention, (**B**) is memory, (**C**) is task switching.

Figure 5A shows the effect of virtual reality exercise technology on attention in minor ADHD patients. The article included six RCT studies, which found (SMD = 0.5, 95% CI (0.03, 0.97), *p* = 0.000, I^2^ = 76.5%) high heterogeneity in the overall effect, which was statistically significant; a random-effects model was used to analyze the effect size. The results of the study showed that the intervention group was superior to the control group in improving attention in minor ADHD patients.

##### Effect of Virtual Reality Movement Technology on Memory in Minor ADHD Patients

Figure 5B shows the effect of virtual reality exercise technology on memory in minor ADHD patients. The article included five RCT studies, which found (SMD = 0.45, 95% CI (0.1, 0.79), *p* = 0.065, I^2^ = 51.8%) moderate heterogeneity in the overall effect, which was statistically significant; a random-effects model was used to analyze the effect size. The results of the study showed that the intervention group was superior to the control group in improving memory in minor ADHD patients.

##### Effect of Virtual Reality Motion Technology on Task Switching in Minor ADHD Patients

Figure 5C shows the effect of virtual reality motion technology on executive function in minor ADHD patients. The article included five RCT studies, which found (SMD = −0.38, 95% CI (−0.72, −0.04), *p* = 0.002, I^2^ = 69.8%) moderate heterogeneity in the overall effect, which was statistically significant; a random-effects model was used to analyze the effect size. The results of the study indicated that the intervention group was superior to the control group in improving task switching in minor ADHD patients.

### 3.5. Sensitivity Analysis

**Figure 6:**
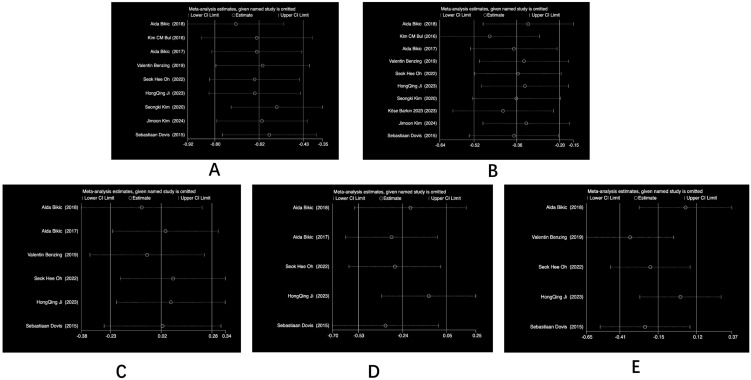
Heterogeneity test: (**A**) cognitive function, (**B**) executive function, (**C**) attention, (**D**) memory, and (**E**) task switching.

In order to explore whether the heterogeneity of the studies was caused by a single study, a sensitivity analysis was therefore carried out on the five outcome indicators, as shown in Figure 6, and the results were all within the 95% confidence intervals, which were robust and did not require the deletion of any literature.

### 3.6. Bias Test

**Figure 7:**
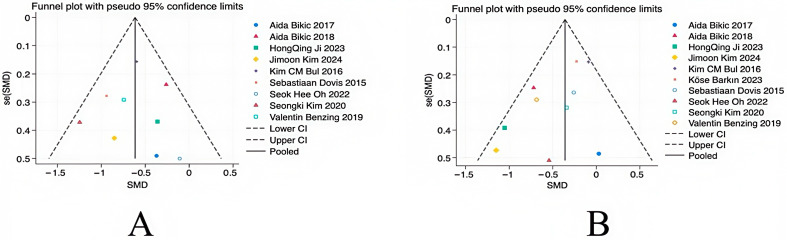
Primary outcome funnel diagram: (**A**) perception and (**B**) execution.

**Figure 8:**
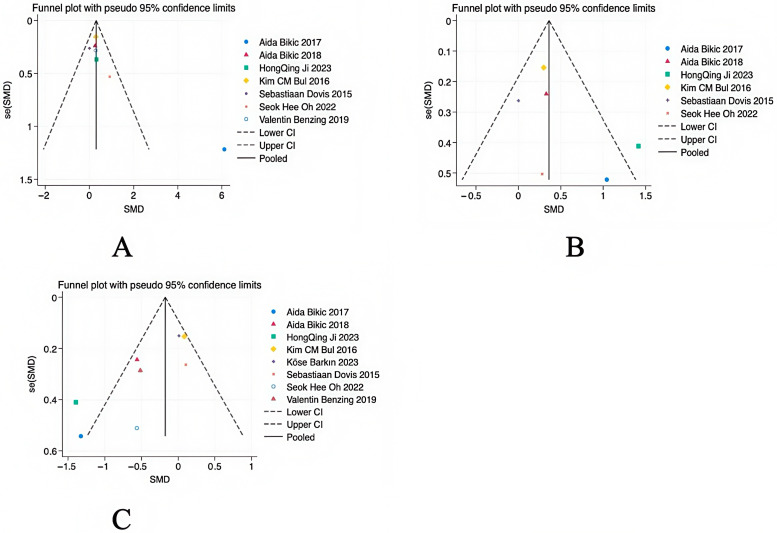
Funnel plot of secondary outcomes: (**A**) attention, (**B**) memory, and (**C**) task switching.

The data of studies on the main outcome variables were included, and a funnel plot was used for the publication bias test. As shown in Figure 7, the results showed a basically symmetrical funnel plot, so it can be judged that there is no publication bias in the literature. When including the data of secondary outcome variables, as shown in Figure 8, the results showed a non-symmetrical funnel plot; it was hypothesized that this problem may be related to the effect size, but it did not affect the final results.

## 4. Discussion

### 4.1. Cognitive Function

#### 4.1.1. Main Outcome Indicators

The results of this study indicate that virtual reality exercise technology has a significant effect on improving cognitive function in children and adolescents with ADHD. These findings align with the studies of Rodrigo-Yanguas et al. (2021) and Oh et al. (2022), both of which demonstrated that virtual reality interventions are effective in enhancing sustained attention and executive function in ADHD patients. Virtual reality motor games, as an emerging therapeutic approach, have proven effective in enhancing executive functions and cognitive abilities in ADHD patients through a multisensory stimulation environment [36,42]. Such interventions enhance sustained attention and task-switching abilities through immersive experiences, thereby improving performance in both academic and daily life contexts, ultimately leading to an improved overall quality of life.

Moreover, the results of this study are consistent with previous research by Liao et al. (2020) [43] and Tuena et al. (2023) [44], both of which highlighted the positive impact of virtual reality on sustained attention and executive function in ADHD patients. These studies reported similar effect sizes, reinforcing the concept that virtual reality can serve as a powerful tool in managing ADHD symptoms. The interactive and engaging nature of virtual reality games not only captures the sustained attention of young patients but also motivates them to actively participate in their treatment—an essential factor in improving adherence to therapeutic interventions. In contrast, earlier studies have shown mixed results regarding the effectiveness of traditional therapeutic approaches for ADHD, which often rely on pharmacological treatments or conventional behavioral therapies. These methods can sometimes lead to limited engagement and motivation among young patients, resulting in suboptimal outcomes. The current study suggests that virtual reality interventions may offer a more engaging alternative, potentially enhancing treatment adherence and overall effectiveness [45].

Additionally, the significant improvement in cognitive function observed in this study, as indicated by the SMD of −0.64, suggests that virtual reality exercise technology not only enhances executive function but may also contribute to improvements in memory and sustained attention, as demonstrated by secondary outcome indicators. The study reported an SMD of 0.5 for sustained attention improvement and an SMD of 0.45 for memory function, both of which indicate substantial positive effects. This multifaceted approach to cognitive enhancement is particularly beneficial for ADHD patients, who often struggle with various aspects of cognitive functioning [46]. In conclusion, the results of this study underscore the potential of virtual reality exercise technology as a novel intervention for improving cognitive function in children and adolescents with ADHD. By fostering engagement, enhancing motivation, and providing a multisensory learning environment, virtual reality can make a significant contribution to better treatment outcomes [47]. Future research should continue to explore the long-term effects of these interventions and their applicability across diverse patient populations, ultimately paving the way for innovative therapeutic strategies in the management of ADHD.

#### 4.1.2. Secondary Outcome Indicators

The results of this study suggest that virtual reality (VR) sports games significantly improve sustained attention in children and adolescents with ADHD. These findings are consistent with those of Graf et al. (2021) and Lis et al., (2016) both of whom demonstrated that virtual reality interventions effectively enhance sustained attention and executive functioning in ADHD patients [48,49]. The engaging and interactive nature of VR technology makes the treatment process more immersive and enjoyable, which effectively stimulates the participation of young patients. In comparison to traditional treatment modalities, VR sports games can better attract patients to actively engage in the treatment, thus improving adherence and, consequently, the overall efficacy of the intervention. This heightened level of participation not only helps patients maintain focus during therapy but also boosts their self-confidence.

Moreover, the significant effect size observed in this study indicates a moderate to large impact of VR sports games on improving sustained attention. This result is particularly noteworthy given the high heterogeneity across the studies included, suggesting that while the overall effect is robust, individual responses to virtual reality interventions may vary. This highlights the need for personalized treatment approaches. In contrast to previous findings, such as those by Pheh et al. (2021) [50], which emphasized the limited efficacy of conventional treatment methods in improving sustained attention, this study underscores the potential of virtual reality as a more engaging alternative. The immersive nature of VR not only captures the sustained attention of ADHD patients but also creates a safe environment in which they can practice focus and task management skills. The implications of these findings extend beyond mere improvements in sustained attention; they suggest that incorporating VR into therapeutic practices could enhance cognitive engagement and motivation among young patients [51]. As such, this innovative approach may not only improve treatment outcomes but also foster a more positive therapeutic environment, ultimately contributing to better overall mental health and well-being for children and adolescents with ADHD.

The results of this study also suggest that VR sports games significantly enhance memory function in children and adolescents with ADHD. These results are consistent with those of De Luca et al. (2024) and Seesjärvi et al., (2022), both of whom found that VR interventions effectively improve memory performance in ADHD patients, particularly in relation to short-term memory during task performance [52,53]. VR sports games can be tailored to each patient’s specific needs and interests, providing a personalized treatment plan. This flexibility allows the intervention to be better adapted to individual patient characteristics, thereby enhancing the treatment’s effectiveness. Different game difficulties and types can be designed for patients of varying ages or with different symptom profiles, thus enabling more precise and targeted interventions.

Furthermore, the moderate effect size observed in this study underscores the potential of VR as a valuable tool for memory enhancement in ADHD patients. Unlike traditional interventions, which often adopt a one-size-fits-all approach, the adaptability of VR games allows clinicians to customize experiences that align with each patient’s unique cognitive profile. This is particularly important given the diverse nature of ADHD symptoms, as some patients may struggle more with short-term memory than others. In contrast, previous studies, such as those by Ahn et al. (2016) [54], have shown limited improvements in memory through conventional behavioral therapies, suggesting that these methods may not adequately address the specific cognitive deficits associated with ADHD. In contrast, the immersive and engaging nature of VR not only captures the sustained attention of patients but also provides a stimulating environment that is conducive to memory retention and recall. The implications of these findings are significant. By integrating VR into therapeutic practices, healthcare providers could enhance cognitive rehabilitation for ADHD patients, leading to improved academic performance and better daily functioning. This innovative approach not only creates a more engaging treatment experience but also empowers patients by equipping them with effective memory strategies that can be applied in various aspects of their lives, ultimately contributing to their overall development and well-being.

### 4.2. Executive Function

#### 4.2.1. Main Outcome Indicators

The results of this study indicate that virtual reality (VR) exercise technology has a significant impact on improving executive function in children and adolescents with ADHD. Upon further investigation, the findings of this study differ somewhat from those of Gillberg et al. (1997) and Hariprasad et al. (2013), who focused on the effects of medication on executive functioning in ADHD patients; their results indicated that medication had a limited effect, failing to significantly improve executive function [55]. In contrast, Hariprasad et al. (2013) examined the impact of traditional behavioral interventions, which showed some effect but did not reach statistical significance [22]. In contrast, our study demonstrates that VR motion technology, as an emerging intervention, can effectively enhance executive function in ADHD patients, offering a promising alternative treatment option. Through the interactive nature of VR-based sports games, patients can cooperate or compete with others, providing them with a safe social environment in which to practice social skills, improve communication and teamwork, and enhance physical coordination and motor skills [39]. For example, in a virtual dance game, patients must follow the rhythm to perform various movements, which not only exercises their physical flexibility but also improves their spatial perception and reaction speed.

Moreover, the significant effect size observed in this study suggests that virtual reality interventions may be particularly effective in addressing the executive function deficits commonly seen in ADHD patients. This finding contrasts with the limited efficacy reported for traditional treatment modalities, indicating that VR offers a more engaging and effective approach. The interactive and immersive nature of VR enables the creation of scenarios that challenge patients to make quick decisions, adapt to changing environments, and develop problem-solving skills, all of which are critical components of executive functioning. Additionally, the results underscore the potential of VR to foster a sense of agency and motivation among ADHD patients. Unlike passive forms of treatment, the active participation required in VR games can lead to increased enjoyment and sustained engagement, both of which are essential for effective learning and skill acquisition. These findings align with those of Nekar et al. (2022) [56], who also reported positive outcomes associated with VR interventions in enhancing sustained attention and executive functioning in ADHD patients.

In conclusion, the integration of VR technology into therapeutic practices not only provides a novel and effective means of improving executive function but also offers a multifaceted approach to treatment that can be tailored to individual patient needs. By enhancing executive functions, VR interventions may ultimately contribute to improved academic performance, better social interactions, and an overall enhancement in quality of life for children and adolescents with ADHD.

#### 4.2.2. Secondary Outcome Indicators

The results of this study suggest that virtual reality sports games have a significant impact on improving task-switching ability in children and adolescents with ADHD. This study differs somewhat from the work of Rodrigo-Yanguas et al. (2021) and Savickaite et al., (2022) as Rodrigo-Yanguas et al. (2021) focused on the improvement of sustained attention through virtual reality interventions, while our study specifically examines task-switching, which is a distinct aspect of executive functioning. This highlights the potential of virtual reality sports games in the realm of multiple cognitive domains [57]. Additionally, although Savickaite et al. (2022) also investigated the effects of virtual reality on ADHD patients, their results did not yield significant evidence regarding task switching, underscoring the uniqueness and importance of our findings [58].

Virtual reality interventions present fewer side effects than traditional medications, offering a safe and effective alternative for patients. Many parents and patients are concerned about the potential for dependency and the side effects of medication, making virtual reality sports games an appealing option for improving sustained attention and executive function without pharmacological intervention, thus alleviating the psychological burden on parents [59]. The significant improvement in task-switching ability observed in this study highlights a critical aspect of executive function that is often impaired in individuals with ADHD. Task switching is essential for cognitive flexibility, enabling individuals to adapt to new information and changing demands. The SMD of −0.38 indicates a meaningful effect, suggesting that virtual reality sports games can effectively engage ADHD patients in activities that require them to shift focus and adjust strategies dynamically. This finding supports the idea that immersive environments, such as those created by virtual reality, can enhance cognitive engagement and facilitate learning through active participation [60].

In comparison, previous studies have yielded mixed results regarding the efficacy of traditional interventions for improving task-switching abilities. While behavioral therapies have demonstrated some benefits, they often lack the engaging elements necessary to maintain the interest of ADHD patients, resulting in suboptimal outcomes. In contrast, virtual reality sports games provide an interactive and enjoyable platform that not only captures sustained attention but also promotes sustained cognitive effort [61]. This level of engagement may be crucial in reinforcing neural pathways associated with task switching, ultimately leading to long-term improvements. Moreover, the ability of virtual reality to simulate real-world scenarios allows patients to practice task switching in a safe environment, where they can make mistakes and learn from them without the pressure of real-life consequences. This experiential learning aspect is vital, as it can enhance the transfer of skills from the virtual environment to real-life situations, thereby improving overall functioning in academic and social contexts. In conclusion, the findings of this study underscore the potential of virtual reality sports games as an innovative intervention for enhancing task-switching abilities in children and adolescents with ADHD. By offering an engaging and effective alternative to traditional treatment modalities, virtual reality can help bridge cognitive gaps, ultimately improving academic performance, social interactions, and overall quality of life for children and adolescents with ADHD.

### 4.3. Limitations of the Article

This article has two limitations, listed as follows. 1. The small number of included studies resulted in high heterogeneity. However, high heterogeneity does not mean that the results are invalid, because heterogeneity between studies is common in many areas of meta-analysis and may instead reflect real differences in effect. 2. The small number of included studies resulted in an overestimation of effect size. The main reason for this is that the number of included studies is small, and the results may not be representative of the entire target population, which may lead to an overestimation of effect size.

## 5. Conclusions

VR motor games provide an effective intervention for improving executive and cognitive functioning in children and adolescents with ADHD. By engaging patients through immersive tasks, customizing treatment, and incorporating real-time feedback, VR enhances sustained attention, memory, and task-switching abilities. This approach offers a promising interdisciplinary method for ADHD treatment, involving both patients’ families and schools.

## Figures and Tables

**Table 1:** Cochrane search strategy.

#1	MeSH descriptor: [Virtual Reality] explode all trees
#2	(Reality, Virtual):ti,ab,kw OR (Virtual Reality, Educational):ti,ab,kw OR (Educational Virtual Realities):ti,ab,kw OR (Educational Virtual Reality):ti,ab,kw OR (Reality, Educational Virtual):ti,ab,kw OR (Virtual Realities, Educational):ti,ab,kw OR (Virtual Reality, Instructional):ti,ab,kw OR (Instructional Virtual Realities):ti,ab,kw OR (Instructional Virtual Reality):ti,ab,kw OR (Realities, Instructional Virtual):ti,ab,kw OR (Reality, Instructional Virtual):ti,ab,kw OR (Virtual Realities, Instructional):ti,ab,kw
#3	#1 OR #2
#4	MeSH descriptor: [Attention Deficit Disorder with Hyperactivity] explode all trees
#5	(ADHD):ti,ab,kw OR (ADDH):ti,ab,kw OR (Attention Deficit Disorders with Hyperactivity):ti,ab,kw OR (Attention Deficit Hyperactivity Disorders):ti,ab,kw OR (Attention Deficit Hyperactivity Disorder):ti,ab,kw OR (Attention Deficit-Hyperactivity Disorder):ti,ab,kw OR (Attention Deficit-Hyperactivity Disorders):ti,ab,kw OR (Deficit-Hyperactivity Disorder, Attention):ti,ab,kw OR (Deficit-Hyperactivity Disorders, Attention):ti,ab,kw OR (Disorder, Attention Deficit-Hyperactivity):ti,ab,kw OR (Disorders, Attention Deficit-Hyperactivity):ti,ab,kw OR (Hyperkinetic Syndrome):ti,ab,kw OR (Syndromes, Hyperkinetic):ti,ab,kw OR (Attention Deficit Disorder):ti,ab,kw OR (Attention Deficit Disorders):ti,ab,kw OR (Deficit Disorder, Attention):ti,ab,kw OR (Deficit Disorders, Attention):ti,ab,kw OR (Disorder, Attention Deficit):ti,ab,kw OR (Disorders, Attention Deficit):ti,ab,kw OR (Brain Dysfunction, Minimal):ti,ab,kw OR (Dysfunction, Minimal Brain):ti,ab,kw
#6	#4 OR #5
#7	#3 and #6

**Table 2:** Basic features of the included studies.

Author	Year	Simple Size (Male/Female)	Source	Age	(T/C)	Intervention Cycle, Frequency, and Duration	Indicators	Measurement Tools
Aida Bikic [32]	2018	70	Denmark	6–13	(35/35)	8 weeks, 6 times/week	cognitive, executive	RVP, SOC
Kim CM Bul [33]	2016	170 (133/37)	Belgium	8–12	(88/82)	10 weeks, 3 times/week, 65 min/time	cognitive, executive	BRIEF
Aida Bikic [34]	2017	17 (12/5)	Denmark	14–17	(9/8)	7 weeks, 5 times/week, 30 min/time	cognitive, executive	RVP, SOC
Valentin Benzing [35]	2019	51 (42/9)	Germany	8–12	(28/23)	8 weeks, 3 times/week, 30 min/week	cognitive, executive	Flanker, EFs
Seok Hee Oh [36]	2022	16	South Korea	8–13	(8/8)	8 times	cognitive, executive	ATA, Stroop
HongQing Ji [37]	2023	30	South Korea	8–12	1 (6/14)	4 weeks, 3 times/week, 50 min/week	cognitive, executive	FAIR, Go/No-go
Seongki Kim [38]	2020	40	South Korea	under 10	(20/20)	15 times in 6 weeks, 50 min/time	cognitive, executive	ATA, IM
Köse Barkın [39]	2023	176	Turkey	7–12	(88/88)	8 weeks, 2 times/week, 45 min/time	executive	BOT
Jimoon Kim [40]	2024	24 (10/14)	South Korea	child	(12/12)	4 times in 8 weeks, 15 min/time	cognitive, executive	ATA
Sebastiaan Dovis [41]	2015	89	The Netherlands	8–12	(28/30)	25 times, 30 min/time	cognitive, executive	stop task, Stroop

**Table 3:** Evaluation results of literature quality and risk of bias of included studies.

Inclusion of Literature (Author)	Random Sequence Generation	Allocation Concealment	Blinding of Participants and Personnel	Blinding of Outcome Assessment	Incomplete Outcome Data	Selective Reporting	Other Bias
Aida Bikic 2017 [34]	L	L	L	L	L	L	L
Aida Bikic 2018 [32]	U	U	L	L	L	L	L
HongQing Ji 2023 [37]	L	U	L	L	H	L	L
Jimoon Kim 2024 [40]	L	L	L	L	L	L	L
Kim CM Bul 2016 [33]	U	U	U	L	L	L	L
Köse Barkın 2023 [39]	L	L	L	L	L	L	L
Sebastiaan Dovis 2015 [41]	L	L	L	L	L	L	L
Seok Hee Oh 2022 [36]	U	U	L	L	L	L	H
Seongki Kim 2020 [38]	U	U	U	L	L	L	L
Valentin Benzing 2019 [35]	L	U	L	L	H	L	L

Note: L: Low risk of bias, U: Unclear risk of bias, H: High risk of bias.

## Data Availability

The data that support the findings of this study are available on request from the corresponding author.
